# Use of non-emergency contraceptive pills and concoctions as emergency contraception among Nigerian University students: results of a qualitative study

**DOI:** 10.1186/s12889-016-3707-4

**Published:** 2016-10-04

**Authors:** Anthony Idowu Ajayi, Ezebunwa Ethelbert Nwokocha, Wilson Akpan, Oladele Vincent Adeniyi

**Affiliations:** 1 Department of Sociology, Faculty of Social Sciences & Humanities, University of Fort Hare, East London, South Africa; 2 Department of Sociology, Faculty of Social Sciences, University of Ibadan, Ibadan, Nigeria; 3 Department of Sociology, Faculty of Social Sciences & Humanities, University of Fort Hare, East London, South Africa; 4 Department of Family Medicine, Walter Sisulu University, Cecilia Makiwane Hospital, East London Hospital Complex, East London, South Africa

**Keywords:** Unplanned Pregnancy, Unsafe abortion, Female university students, Emergency contraception, Concoctions, Misconceptions, Danger, Efficacy, Unprotected sex, Risky Sexual behaviours

## Abstract

**Background:**

Emergency contraception (EC) can significantly reduce the rate of unintended pregnancies and unsafe abortions especially in sub-Saharan Africa. Despite the increasing awareness of EC among educated young women in Nigeria, the rate of utilisation remains low. This study therefore explores the main barriers to the use of EC among female university students by analysing their knowledge of emergency contraception, methods ever used, perceived efficacy, and its acceptability.

**Methods:**

This paper brings together the findings from several focus groups (*N* = 5) and in-depth interviews (*N* = 20) conducted amongst unmarried female undergraduate students in two Nigerian universities.

**Results:**

Participants considered the use of condom and abstinence as the most effective methods of preventing unplanned pregnancy. However, many participants were misinformed about emergency contraception. Generally, participants relied on unconventional and unproven ECs; Ampiclox, “Alabukun”, salt water solution, and lime and potash and perceived them to be effective in preventing unplanned pregnancies. Furthermore, respondents’ narratives about methods of preventing unwanted pregnancies revealed that inadequate information on emergency contraception, reliance on unproven crude contraceptive methods, and misconception about modern contraception constitute barriers to the use of emergency contraception.

**Conclusions:**

The findings suggested that female university students are misinformed about emergency contraception and their reliance on unproven ECs constitutes a barrier to the use of approved EC methods. These barriers have serious implications for prevention of unplanned pregnancies in the cohort. Behavioural interventions targeting the use of unproven emergency contraceptive methods and misperceptions about ECs would be crucial for this cohort in Nigeria.

## Background

Emergency contraception is the use of a drug or device to prevent pregnancy [1]. It could be used after an unprotected sexual intercourse, contraceptive failure, and coerced unprotected sex or in cases of sexual assault. Studies have shown that the rate of unplanned pregnancy is very high worldwide [2] and unwanted pregnancy is the main reason women seek abortion [3]. Emergency contraception could be very crucial in preventing unplanned pregnancies in settings where abortion is illegal, however, following the evidence presented in the literature, emergency contraception has not been demonstrated to have a population level impact with regards to the reduction in the rate of unplanned pregnancies [4, 5]. Globally, many unplanned pregnancies end up being aborted in safe and unsafe conditions [6]. In sub-Saharan Africa, over half of unplanned pregnancies are aborted in unsafe conditions leading to deaths and serious complications [7]. Abortion-related mortality accounts for an estimated 8 % of global maternal deaths [8]. Distressingly, half of the abortion-related mortality occurs among young women in sub-Saharan Africa [7]. This clearly suggests that young women as a demographic segment have a special need for emergency contraception.

The question then is why has emergency contraception remained underutilised despite its availability for over 40 years? Many studies have reported various reasons for low level of EC utilisation and these reasons vary by context [9–12]. There seems to be a consensus among scholars that a lack of awareness restricts access to the use of EC. Similarly, increased access to emergency contraceptive pills was associated with greater use [13]. However, it is not clear why emergency contraceptive pills have not been shown to reduce unintended pregnancy rates at a population level despite increased availability and unrestricted access.

A review of studies on EC among young women in Nigeria shows that the rate of awareness of EC varies from as low as 29 % [14] to as high as 73 % [15]. The rate of awareness of EC reported varies by context, conceptual definition of EC and demographical composition of participants. While some studies define EC as method of preventing unplanned pregnancies after sex [15–17], which gives room for the mentioning of unconventional EC methods and consequently exaggerated level of awareness of EC, other studies specifically focused on conventional EC methods with slightly lower levels of awareness of EC. Studies that provide names of EC options for participants to choose from often report a higher proportion of awareness of EC. Likewise, a study that has high proportion of sexually active participants tend to report high rate of awareness of EC [14].

Similarly, the use of EC varies from as low as 13 % [18] to as high as 52 % [14], however, the reported level of use of EC varies by demographic composition of participants, context, and differences in EC conceptualisation. One thing is clear, the reported level of awareness of EC in most studies is much higher than the level of use in those studies. The inconsistency in the level of use in similar settings clearly indicates the need for further study to explain the results and possibly identify impediments to the use of EC. When does awareness of EC translate to its use? When does an individual who is unaware of EC become conscious of EC methods and subsequently utilise EC? These are grey areas in the discourse of EC in Nigeria and this could serve as input for policy makers when crafting interventions for prevention of unplanned pregnancy.

Most studies on EC adopted quantitative design, which does not allow for in-depth probing and understanding of the phenomenon. Also many of the studies on EC are strong from empirical perspectives but weak from a theoretical viewpoint. This is not to say that these studies are not informed by theories. Nonetheless, those studies do not allow for theorising on the use of EC. Using the constructs in the health belief model [19] and from a qualitative paradigm, this study explores the barriers to the use of emergency contraception among female university students by analysing their knowledge of emergency contraception, methods ever used, perceived efficacy, and its acceptability.

## Methods

### Settings

The study was conducted in Ekiti State University and Afe Babalola University both in Ekiti State, South Western Nigeria, between the months of February and April, 2012. Afe Babalola University, located in Ado-Ekiti, is a privately-owned university established in 2009 and has five colleges: Science, Law, Social and Management Sciences, Engineering, and Medicine and Health. It is largely a residential university that accommodates about 900 female students. Ekiti State University (formerly, University of Ado-Ekiti), was established in 1982 and has nine faculties (colleges): Arts, Agricultural Sciences, Education, Engineering, Law, Management Sciences, Sciences, Social Science and College of Medicine. The university has a limited number of on-campus residential facilities, which accommodate over 12,000 students. Many students live in rented apartments in the towns of Ado-Ekiti and Iworoko-Ekiti. The university has a female student population of 5840.

### Participants

Participants were unmarried female students in their first to fifth year of study. A total of 56 participants took part in the study, 20 and 36 in the in-depth interviews and focus group discussions, respectively. The sociodemographic characteristics of the participants are summarised in Table 1. The participants were aged between 17 and 28 years.

**Tables 1:** Characteristics of respondents

Characteristics	In-depth Interviews (*n* = 20)	Focus group discussions (36)
Age		
17–19 20–22 23 and above	6 (30) 8 (40) 6 (30)	12 (35.3) 13 (38.2) 9 (26.5)
Year of Study		
First Second Third Fourth Fifth	4 (20) 5 (25) 6 (30) 3 (15) 2 (10)	9 (26.5) 8 (23.5) 8 (23.5) 9 (26.5) - (−)

### Study design

A total of five focus group discussions and 20 in-depth interviews were conducted with participants selected from the two universities. The respondents were recruited in February and April 2012. Participants were purposively selected in both universities. A pre-piloted semi-structured interview guide was utilised to obtain qualitative data from each participant. The interview guide covered the following topics: questions regarding the methods of prevention of unplanned pregnancy before and after sexual intercourse, methods ever used and efficacy of those methods, perceived efficacy and side effects of using pills and acceptability of EC [19].

The in-depth interview sessions lasted between 45 and 60 min. A trained research assistant conducted the interviews in English. The interview sessions were audiotaped and field notes were taken. Recruitment of informants and interim analyses were conducted after every five interviews until no new information emerged during the interviews (data saturation). In total, 20 IDI Participants (13 from Ekiti State University and seven from Afe Babalola University) were involved in the study.

Focus group discussions (FGDs-five in total) were used as a complementary data collection method. Each FGD included six to eight (unmarried) female undergraduate students purposively selected from the different levels of study. An FGD session lasted approximately 60 min. All discussions were audiotaped. Field notes focused on capturing not only verbal but also non-verbal cues of the participants. In total 36 female students participated in the various FGDs.

### Ethical consideration

The university of Ibadan Social Sciences and Humanities ethic committee approved the study. Voluntary participation, informed (verbal) consent, confidentiality, and respondent anonymity were strictly observed. The inclusion of participants was voluntary and verbal consent was obtained from each participant. Participants were guaranteed confidentiality and anonymity. Furthermore, participants were assured that the research findings were for academic use only. Overall, the study was conducted in line with the research ethics policy of the University of Ibadan.

### Data analysis

The qualitative data obtained through the in-depth interviews and FGDs were transcribed verbatim and coded, with the application of Atlas 6.2 software. Additional information was obtained from the field notes. Thematic content analysis was conducted on the qualitative data. Notes were read several times and responses of participants were organised. The organised data was then subjected to content and narrative analyses. The first stage of analysis was limited to grouping data into issues that deal directly with the objectives of the study.

Thematic content analysis was employed to maximise the chance of all relevant information being grouped and coded appropriately. The notes were crosschecked to ensure responses of participants were grouped appropriately. In the second stage, further analysis was carried out to explore sub-themes and unanticipated issues. The third stage involved a critical and reflexive review of interviews and FGD in order to construct a narrative on how young women went about preventing unwanted pregnancies. To further enhance data credibility, a random sample of five interviews (including audiotapes, field notes and coding of themes) was carefully reviewed by two of the authors before the data collection cycle came to a close, and issues picked up were discussed and addressed by the research team members before the data collection cycle came to an end.

## Results

The main findings of the study are presented below under themes that mirror the study’s objectives.

### Perceived susceptibility to unplanned pregnancy

Participants not in a sexual relationship mentioned that they were not susceptible to unplanned pregnancy because they were practicing abstinence while those in sexual relationships believed that the use of contraceptives has significantly reduced their risk of getting pregnant. To many, preventing unplanned pregnancy is as simple as expressed by this participant;

*You either stay away from sex or protect yourself (23 year old third year student).*

Nonetheless, some participants acknowledged that unplanned pregnancy is still a risk they face because the use of contraception does not guarantee a 100 % prevention of unplanned pregnancy. Respondents generally believed that broken condoms, missed pills or outright neglect of condom use could make them susceptible to unplanned pregnancies. Their narratives suggest that sometimes a condom could break without the knowledge of the female; however if their partner informs them they could take some drugs to prevent unplanned pregnancy. One participant indicated that she practices after sex contraception because she believes her partner may deliberately not inform her.

*Sometimes, you may not know that the condom burst especially if the man does not tell you (IDI, 22 years old year three student).*

### Perceived severity of unplanned pregnancy

Most participants consider unplanned pregnancy to have many negative consequences. The perceived stigma from friends, alienation from parents, perceived effect on education and perceived stringent financial implication were mentioned as reasons why most participants consider unplanned pregnancy to be severe. The views from some of the study’s participants capture this:

*My parents are disciplinarians. My Father can disown me as a daughter if he hears that I got pregnant in school seeing how stressful it is to pay my school fees (IDI, 23 year old fourth year student).*

*If I get pregnant, I may not be able to cope financially. My parents would not support me if I get pregnant in school because they sent me to learn in school and I am meant to behave myself (FGD, 18 year old, second year student).*

*It is like a stigma to get pregnant in higher institution. People have a negative perception towards pregnancy out of wedlock. The Yoruba’s would say that “ko l’oyun, o gb’oyun ni” (A term used to express disapproval of premarital unplanned pregnancies). Your classmates would think that you are not smart enough (22 year old, fourth year student).*

Many participants stated that they go the extra mile in their quest to prevent unplanned pregnancy with some even reporting having a history of abortion.

### Method of preventing unplanned pregnancy

Research participants expressed strong support for abstinence and the use of condoms, referring to these as not only the safest forms of contraception but as the most efficacious ways to prevent the consequences of unplanned pregnancies. Most of the respondents in their early years of study particularly favoured abstinence, and these respondents were also the least knowledgeable about contraceptive issues. Among the sexually active participants (*n* = 43), especially those in the later years of study, male condom was the preferred contraceptive.

Overall, respondent’s narratives revealed three main contraceptive use patterns, namely, condom only, condom or pills, and condom, pills or douching. One respondent remarked thus about her use of the first pattern:

*“I use protection, condoms of course; I always insist that my partner use a condom, because I do not think that a female condom is hygienic enough”. (IDI, 25-year old, fourth-year student).*

The second pattern, the use of either condom or emergency pills, was evident in the following response:

*I protect myself by using condoms or pills. I use Postinor* 1 *(IDI, 20-year old, year three student).*

The third pattern, condom, pills or douching, was summed up by a 24-year old respondent:

*I use condom, but after sex I use drugs-Postinor - or salt and water or wash off immediately. (FGD, 24-year old, year four student).*

### Methods of preventing unplanned pregnancies after-sex

Most participants indicate some methods of preventing pregnancies after sex although not all methods mentioned are conventional. Respondents who did not have any knowledge of after sex contraception were relatively young and mainly in their first year of study. The study revealed that students also relied on what was locally termed “concoction” – a mixture of substances with unproven efficacy, such as salt and hot water, soft drinks, a local brand of analgesic known as *Alabukun*, lime and potash, and lime and *Alabukun.* Respondents were convinced that these concoctions were highly efficacious as after-sex contraceptives:

*If unprotected sex happens, instantly, there are some drugs like lime and “Alabukun” at the same time; they work in most cases. (IDI, 24-year old, fifth-year student).*

*The local methods work. I have used salt and water* 2 *to evacuate the sperm. It works. (FGD, 20 year old, year three student).*

For one younger participant, her belief in the efficacy of “contraceptive” concoctions was based on “medical” authority”

*A nurse once told me that you can drink the mixture of cold “7UP” [a brand of carbonated drink] and “Alabukun”* 3 *to wash the womb (IDI, 18 year old, year one student).*

Beside concoctions, some participants said they could personally testify to the efficacy of certain non-emergency contraceptive drugs. According to them, menstrogen tablets^4^, antibiotics^5^, and gynaecosid tablets^6^ were particularly efficacious. Some participants were not only knowledgeable about approved emergency contraception pills (Levonorgestrel (Postinor), for example) but had personally used them.

*I have used Postinor and it works (FGD, 23 years old, year four student).*

The study found that in their quest to prevent unwanted pregnancy, students also relied on combinations that could prove quite dangerous, such as combining non-emergency contraceptive pills with what was described earlier as “concoctions”. One 22-year old respondent said that such a combination eliminated any chance of pregnancy after unprotected sex:

*Menstrogen first, then lime and potash, can be used to prevent unwanted pregnancy. I have used this combination and it works (FGD, 22 years old year three student).*

For some respondents, what worked best was a combination of emergency contraceptive pills with non-emergency contraceptive pills. As one 23-year old fourth-year student explained, EC pills such as Postinor I, Postinor 2 “worked better in combination” with non-EC drugs such as ampiclox capsules (antibiotics) and menstrogen.

Some participants preferred the combination of ECPs and concoctions:

*I will use Postinor immediately, and then use very hot water and plenty of salt-just to be sure. (FGD, 21-year old, third year student).*

Also as an after-sex contraceptive practice, participants reported that the method that worked for them was manual extraction of the semen through vaginal douching immediately after intercourse:

*I stand up immediately and go the bathroom and try to bring out the sperm. (FGD, 28-year old fourth-year student).*

*You can use even Summer’s Eve Douche* 7 *[a brand of vagina wash]. It would flush out the sperm. That is what I use. (FGD 26-year old fourth-year student).*

While these various patterns of after-sex contraception have side effects, respondents’ narratives revealed that such side effects had become wrapped up in misconceptions and myths, even among the undergraduate students. Some of these misconceptions are illustrated in the next section.

### Perceived efficacy and side effects of using emergency contraceptive pills

Firstly, the study found that some participants have doubts about the efficacy of medically approved ECPs, are exaggerating the side effects of ECPs and are associating it with infertility:

*Too much of Postinor can cause damage to one’s womb, and one may not be able to fall pregnant again. (IDI, 20-year old, third-year student).*

One 18-year old student’s preference for condoms was informed by her distrust of contraceptive pills:

*I prefer condom, as the use of drugs are not good. It may not work and it can negatively affect the woman’s body (IDI, 18 year old, year one student).*

Lastly, while the link between EC and future miscarriage is not documented as an EC complication, respondents assumed this link:

*Too much of postinor-2 will weaken the wall of the womb and damage the uterus. This will cause miscarriages in the future. (IDI, 25-year old fifth-year student).*

### Acceptability of emergency contraception

Despite the apprehension of few participants about the side effects of EC, almost all participants indicated that they would use EC if faced with the risk of unplanned pregnancy.

*“I would be scared of pregnancy risks and I would use the contraceptives like Postinor (FGD, 20 year third year student).*

Even respondents who do not have knowledge of EC indicated their readiness to make use of EC if faced with the risk of unplanned pregnancies. Some indicated that they would consult a medical practitioner and the viewpoints of two students, one in year one and the other in year two, are thus presented below;

*I can ask for advice from the pharmacy or anywhere (IDI, 19 year old, year one student).*

*I would go to the hospital, as they are experts. I cannot use pills as the pills can cause more problems. I have a friend that used pills, which caused her a lot of problems (FGD, 21 year old, second year student).*

Most participants expressed a general positive attitude to the use of EC although many would rather use condoms or abstain from sex altogether.

## Discussion

Most studies on EC adopted a quantitative approach which limits the in-depth understanding of methods adopted by young women to prevent unplanned pregnancies and barriers that hinder them from utilising approved emergency contraception. The main contribution of this qualitative study is the in-depth analysis of the perceptions of female university students about their susceptibility to and severity of unplanned pregnancies, knowledge about methods of preventing unplanned pregnancies and barriers to the use of EC.

The findings of this study underscore the relevance of health belief model in understanding preventive health behaviour and also allow us to theorise on the use of emergency contraception. Our results on perceived susceptibility to unplanned pregnancy show that many female students are susceptible to unplanned pregnancy due to non-utilization of contraceptives [17, 20–28] and contraceptive failure. However, many of them may not realise that they are susceptible to unplanned pregnancy, and as such, would not take preventive action such as the use of emergency contraception. A study conducted among women undergoing induced abortion reported this as part of the barriers to the use of EC [29]. Timely identification of perceived risk of unplanned pregnancy, ‘most especially in cases of broken condom’ is an important condition for the use of EC.

Our findings on perceived severity of unplanned pregnancy clearly suggest that the chances of the use of EC for young women with perceived risk of unplanned pregnancy is high. Many participants considered unplanned pregnancy to have high severity and would try any available methods including abortion to avoid it.

As suggested by the health belief model [19], awareness and knowledge of EC are important factors that could influence the use of EC. Extant studies on EC reported considerable high level of awareness and low level of use of EC. Inferring from our study, it appears that many students only become aware of EC when the need for their use arises, that is, when they become sexually active or have a perceived risk of unplanned pregnancy. Our findings suggest that in most cases sexual activities precede the knowledge of emergency contraception. As such, desperation to prevent unplanned pregnancy predisposes female university students to try unproven and unapproved emergency contraception.

This study reveals that most students have some knowledge about methods of preventing unplanned pregnancies prior to and after sex, however, some of the methods mentioned are unconventional. Many of the pills reported by the participants, as emergency contraceptive pills were, in fact, non-ECPs and clearly indicate that they were misinformed. The use of non-EC Pills (gynaecosid, Menstrogen, Antibiotics) and apparently dangerous portions of analgesics^8^^,^ 9, salt, water, caustic potash, carbonated drinks have been reported in similar studies in Southern Nigeria and some parts of Ghana, [16, 17, 30–36] and appear to be in common use among this cohort. Menstrogen (an abortion pill), gynaecosid (a pill for irregular menstrual cycle) and antibiotics (medications for bacterial infections) are not proven ECs, as such; their use mostly without prescription could result into adverse drug abuse and its complications if left unchecked.

The consensus by many respondents that concoction and non-EC pills prevent someone from getting pregnant could well have been reached based on poor knowledge of the ovulation cycle; that is, the fact that sexual intercourse might have taken place before or after the period of ovulation. In other words, pregnancy could not have occurred at all. Some of the practices may in fact be harmful, for instance vaginal douching has been found to be a harmful practice. A number of studies have associated vaginal douching with adverse gynecological and pregnancy-related outcomes as well as sexually transmitted infections (STIs) [37–41].

Although this study confirms extant literature on myths and misconceptions about contraceptive pills and emergency contraceptive [21, 24, 42–44], our findings on acceptability of EC, suggest that many participants would use EC pills to prevent unplanned pregnancy if there is perceived risk of unplanned pregnancy and provided they have the knowledge of EC irrespective of the reported myths and misconceptions. Nonetheless, efforts must be geared towards dispelling the myths and misconceptions about emergency contraception. Previous studies had reported moderate to high level of acceptability of EC [45, 46].

The findings from this study also shed some light on barriers to the use of emergency contraception among female university students. Our study shows that inadequate knowledge about emergency contraception most especially among younger and possible sexually inactive students constitute a major barrier to preventing unplanned pregnancy after sex. It also suggests that the high rate of unplanned pregnancies and consequent abortion among young women is not due to lack of trying to use EC, however, the use of inappropriate methods may have undermine their efforts. This may explain why emergency contraception has not made the required population level impact in preventing unplanned pregnancy.

### Limitations of the study

The qualitative findings reported in this article do not allow for generalization to other populations. They do, however, lay a foundation for future studies, which should involve male students and policy makers,, and also estimate the proportion of opportunities lost to prevent unplanned pregnancy due to the use of non-EC methods. However, the wider distribution of participants across age groups and year of study as well as the multi-method approach adopted for this study did lend credibility to the findings. The in-depth probing of knowledge of prevention of unplanned pregnancy among our participants allowed for deep understanding of the main barriers to the use of EC in our study settings and by extension in some sub-Saharan African countries.

From a policy point of view, the understanding of young women’s perceptions and perspectives vis-a-vis the methods for preventing unwanted pregnancies must be taken seriously in designing interventions. Discouraging the use of non-approved EC should feature mainly in any intervention on preventing unplanned pregnancy among university students.

## Conclusion

The findings suggested that female university students are misinformed about emergency contraception and their reliance on unproven ECs constitutes barrier to the use of approved EC methods and also, have serious implications for prevention of unplanned pregnancies in the cohort. Behavioural interventions targeting the use of unproven emergency contraceptive methods and misperceptions about ECs would be crucial for this cohort in Nigeria.

## Abbreviations

EC: Emergency contraception
ECPs: Emergency contraception pills
FGDs: Focus group discussions
IDIs: In-depth interviews
STDs: Sexually transmitted diseases
STIs: Sexually transmitted infections

## Footnotes

1 Postinor is the brand name for emergency contraceptive pill in Nigeria. It is available over the counter without prescription and the main ingredient is levonorgestrel.

2 Salt and Water commonly used as emergency contraception by our study group said to be taken within 5 min of unprotected sex has not been shown by any study to be effective in preventing pregnancy.

3 Alabukun is a local analgesic

4 Menstrogen is originally a medication made to induce menstration.

5 Antibiotics such as ampiclox are anti-bacterial medications.

6 Gynaecosid is an irregular menstral cycle pill.

7 Vagina Douching is a practice of cleaning the vagina with soap and water or other materials

8 Lime and Potash.

9 Alcoholic drinks
